# An elementary form of agency at planetary-scale

**DOI:** 10.1038/s41598-026-61878-9

**Published:** 2026-08-03

**Authors:** Sergio Rubin, Conor Heins, Takahito Mitsui, Lancelot Da Costa, Karl Friston

**Affiliations:** 1 Vrije Universiteit Brussel, CLEA, Brussels, Belgium; 2 Centro Nacional de Investigaciones Biotecnologicas, CNIB, Cochabamba, Bolivia; 3 Department of Collective Behavior, Max Planck Institute of Animal Behavior, Konstanz, Germany; 4 Faculty of Health Data Science, Juntendo University, Chiba, Japan; 5 ELLIS Institute, Tübingen, Germany; 6 Wellcome Centre for Human Neuroimaging, University College London, London, UK

**Keywords:** Climate homeorhesis, Non-equilibrium steady-state (NESS), Gaia hypothesis, Autopoiesis, Free energy principle, Synchronisation map, Biological autonomy, Climate sciences, Ecology, Ecology

## Abstract

The claim that climate homeorhesis—non-equilibrium steady-state dynamics that underlie Earth’s long-term habitability—depends on an integrated biosphere–climate system capable of acting on its own is central to debates surrounding the Gaia hypothesis. This paper presents a proof of concept suggesting a minimal form of planetary agency based on a simple biosphere–climate system model operating through active inference. This means that the internal states of the system (*biosphere*) will appear to infer shifts in net incoming solar radiation (*external states*) through its *boundary states*, i.e. by integrating past and current surface temperatures (*sensory states)* and actively changing greenhouse forcing (*active states*) to maintain climate trajectories within geophysiological bounds. When surface temperature is altered by solar fluctuation changes, the system adjusts greenhouse and albedo parameters to restore expected climate trajectories, analogous to correcting sensory prediction errors in biological systems. Perturbing active states (e.g., imposed changes in albedo) does not reduce inference precision. Inference robustness decreases when the system’s degrees of freedom increase. Active inference falls, and thus climate homeorhesis fails when there is no biosphere activity (lifeless planet) or when it is decoupled from the climate system. Predictive behaviour operates within the range of solar constants consistent with life’s presence on Earth (~ 4 Ga to ~ 1.5 Ga expected in the future); outside this range, inference fails, undermining a putative form of planetary agency.

**Supplementary Information:**

The online version contains supplementary material available at https://doi.org/10.1038/s41598-026-61878-9.

## Introduction

A lengthy review of the geological evidence suggests that the Earth’s surface temperature has remained within 0–40 °C since the Archean era (4 Ga)^1^, despite an early weak solar luminosity^2^. More specifically, from the Archean to the beginning of the Proterozoic (~ 2.5 Ga) solar luminosity was 30–20% weaker than at present, and the Sun was only 76–83% as intense as it is today^3^. At this lower level of solar luminosity and in a lifeless Earth with the current atmospheric greenhouse gas-volume fraction, composition, and other global parameters held constant, the Earth’s mean temperature would have been well below the freezing point of seawater (− 18 °C), oceans would have been frozen until 1 Ga (when multicellular life already existed on Earth), with glaciers reaching the Equator^4–6^. But the presence of sedimentary rocks and evidence from detrital zircons suggests the existence of liquid water and oceans on the Earth 3.8 Ga and 4.4 Ga respectively^7^ and hence the presence of active planetary life. With increased solar luminosity, the opposite effect can also occur: an increase in Earth’s temperature, evaporation of the oceans, and a runaway greenhouse effect leading to a hot desert state^8–10^.

The proposition that life promoted the long-term homeostasis of Earth’s surface temperature—or more precisely *homeorhesis*, the term introduced by C. H. Waddington to describe a system’s tendency to return to a characteristic trajectory after perturbations or under stochastic noise^11^—has crystallised in the formulation of the Gaia hypothesis^12–15^ and the establishment of Earth system science^16^. Two main biogenic processes have been proposed to be linked to climate homeorhesis, i.e., *Non-equilibrium steady-state* (NESS) dynamics, notwithstanding the faint young Sun and then the gradual increase in solar luminosity over geological time^17–19^. The first one appeared to offset the faint young Sun and its lower radiation^5,6,20^ via the biogenic production of higher carbon based gas-volume fraction such as methane^21–23^—and hence higher greenhouse effect^24^, but reduced albedo—, and the second one counteracting warming via damping greenhouse gas concentrations and increase in albedo surface as solar output intensified^17–19^. A biogenic sulphur-based aerosol-climate linkage has been postulated, through the albedo effect of clouds to counterbalance the rise in incoming solar radiation, hence Earth’s surface temperatures that would otherwise result from the increase in solar luminosity output^25–28^.

Building on this, we have previously formalised the Gaia hypothesis suggesting that the Earth could be considered with life-like properties and thus maintaining geophysiological bounds insofar as its dynamics can be mapped onto an autonomous active inference process^29–33^. Here, we implement this formulation in a biosphere–climate dynamical system model and show that it engages in active inference of shifts in net incoming solar radiation to sustain homeorhetic, NESS climate dynamics. This inference process operates through active adjustments in greenhouse gas concentrations and surface temperature, leading to an elementary form of biological autonomy on a planetary scale.

## Biosphere-climate model structure

**Fig. 1:**
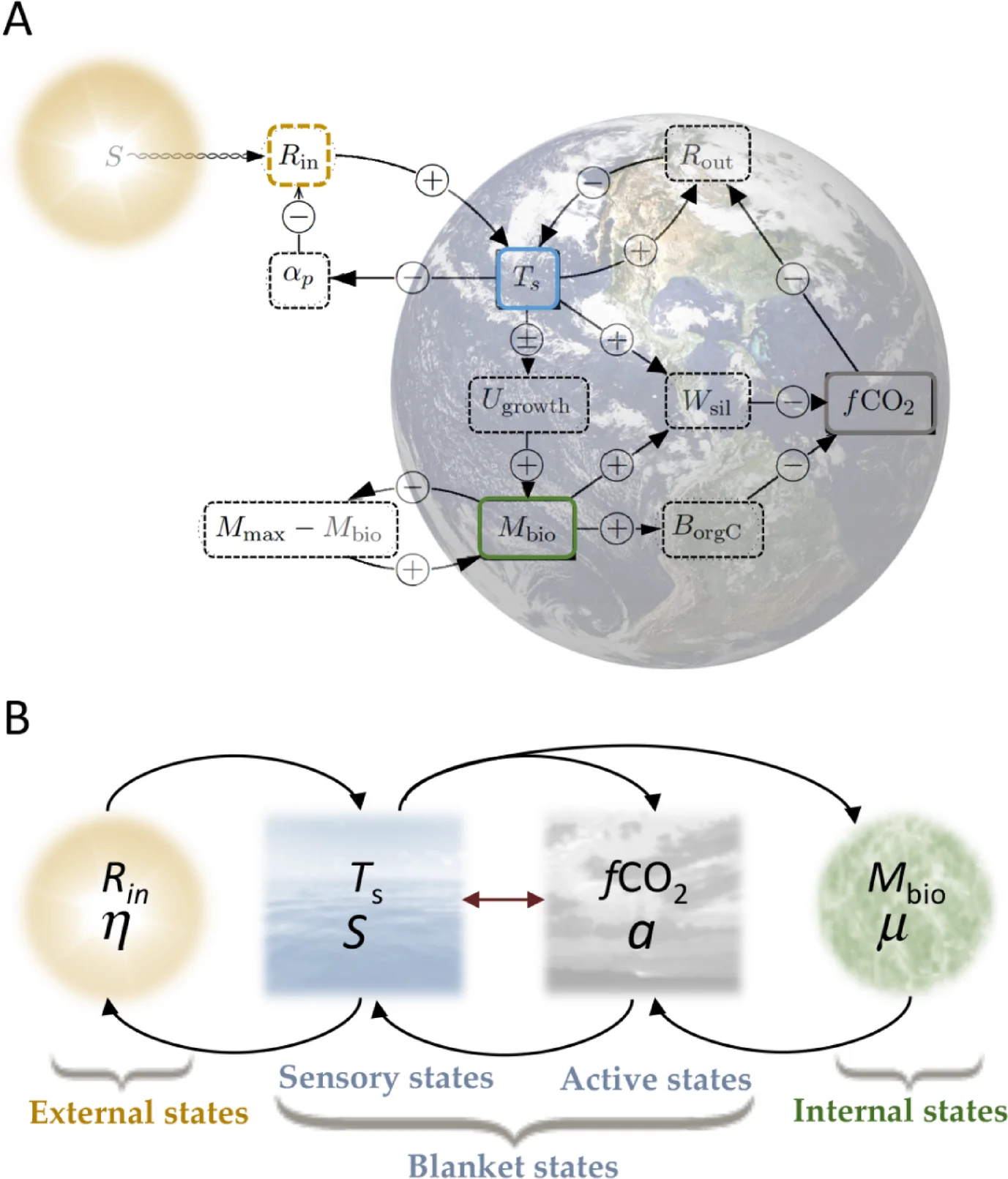
A model of Earth’s biosphere–climate interaction with solar input through boundary/blanket states.** (A)** Schematic of Earth’s biosphere–climate deterministic model. Solid boxes (e.g. blue, green and grey) represent prognostic variables, while dotted boxes (e.g. yellow and black) represent diagnostic variables. Yellow, blue, grey and green boxes represent the net incoming solar radiation (*R*_*in*_), surface temperature (*T*_*s*_), atmospheric mixing ratio of carbon dioxide (*fCO*_*2*_) and the Biosphere biomass (*M*_*bio*_) respectively. Arrows indicate the web of causal relationships between the variables. **(B)** Active inference model of biosphere–climate interaction with net incoming radiation depicting the partitioning of the system into internal, boundary (blanket) and external states. The *T*_*s*_ and *fCO*_*2*_ represent the sensory (*s*) and active (*a*) states respectively–which are the boundary states–while *M*_*bio*_ and *R*_*in*_ represent internal (*µ*) and external (*η*) states. The colours of the graphic of each state (*s*,* a*, *µ*, *η*) are the same as the colours of the boxes in A. The arrows preserve the same causal relationships between states variables as in A.

In our analysis, instead of focusing on the interaction between the biosphere and climate^34,35^, we focused on the interaction between the entire biosphere-climate system with the net incoming solar radiation (Fig. 1A). We applied formal arguments and a tractable method from active inference, also known as Free Energy Principle (FEP) or predicate coding^36,37^ to demonstrate the plausibility of life-like properties, and thus predictive behaviour of the Earth system.

Active inference is a dynamical formalisation of autopoietic embodied cognition (agency)^31^, which postulates that living systems, due to their self-producing organisation^38^, actively predict their environment to maintain their physiological states within a homeorhetic range^39^. Such active inference means that quantities internal to the system (i.e. internal states $\:\mu\:$) can be described as continuously inferring quantities external to it (i.e. external states $\:\eta\:$), given information at the system’s boundary or blanket states (*b*). The boundary comprises a set of sensory (* s*) and active (* a*) states, wherein active states influence but are not influenced directly by external states, and sensory states influence but are not influenced directly by internal states^31^. In other words, the boundary (sensory and active states) intervenes between external and internal states, such that all interactions between the system and its environment occur via the boundary. The stochastic dynamical equations that form the basis of this treatment can be summarised as follows:

$$\,\mathop {\mu \,}\limits^{.} = f_{{\mu \,}} \left( {s,a,\mu \,} \right) + \omega \,_{{\mu \,}}$$

$$\,\dot{a} = f_{a} \left( {s,a,\mu \,} \right) + \omega \,_{a}$$

$$\,\dot{s} = f_{s} \left( {\eta \,,s,a} \right) + \omega \,_{s}$$

$$\,\mathop {\eta \,}\limits^{.} = f_{{\eta \,}} \left( {\eta \,,s,a} \right) + \omega \,_{{\eta \,}}$$

Where the functions * f* are the flow equations and the processes $\:{{\upomega\:}}_{i}$ denote statistically independent random fluctuations. Thus, in formal terms the system’s dynamics can then be formulated as performing active inference, wherein internal states encode a posterior probability density (a.k.a., Bayesian belief) $\:{q}_{\mu\:}\left(\eta\:\right)$ about external states, and active states continuously use this information to regulate sensory states^40,41^. Moreover, these beliefs (posterior probability density) are continuously updated in the face of new signals (information) from the boundary,

$$q_{{\mu _{t} }} \left( \eta \right) \approx p\left( {{{\eta }}_{t} | b_{t} } \right)$$

so as to track evolving external conditions and thereby predict sensory states, such that active states can act on external states to realise the predictions. The implicit circular causality can be read as an action-perception loop^42^ that describes the vicarious exchange between internal and external states, via the boundary (sensory and active) states (Fig. 1B).

A stochastic dynamical model of Earth’s biosphere–climate system interacting with net incoming solar radiation was derived from a deterministic model^34,35^ including comprehensive treatments of the long-term biosphere-climate interaction based on geological climate records^9,43,44^. The deterministic model implements realistic ice-albedo and greenhouse effects (instead of Daisy colours as in Daisyworld model)^45^ where albedo is the biosphere-climate mediator. It features a special focus on the biological causes of the atmospheric content of carbon dioxide, and thus the greenhouse effect, and how it affects the dynamics of Earth’s surface temperature, pointing toward a explanation of one of Earth’s extreme glaciations, rather than climate stabilisation. It entails physically plausible equations of energy balance for the Earth’s surface temperature *T*_*s*_ (K), the long-term carbon cycle for the atmospheric *CO*_*2*_ mixing ratio (the volume fraction of *fCO*_*2*_), a logistic growth model for the biosphere (Biomass, $\:{M}_{bio}$), and the net incoming solar radiation ($\:{R}_{in}$). The state of the ocean, atmosphere and ice-caps is given as a function of *T*_*s*_. The biosphere is parametrized by a relative global mean^34,35^. A logistic growth representation governs biomass, i.e., the treatment of the biospheric component is similar to Daisyworld^45^. The atmospheric *CO*_*2*_ mixing ratio is governed by a simple long-term carbon cycle model that considers the biomass-dependent organic carbon burial, the temperature-dependent silicate weathering and the volcanic outgassing^34,35^. The functional form of the deterministic model is shown as black arrows and boxes in Fig. 1A. See its dynamical multivariate ordinary differential equations [ODE Eqs. 1–4] and parameter values (Table 1) in our methods section.

To entail active inference at planetary level, we established a state partition based on the conditional independencies present in the deterministic model. Thus, biomass logistic growth $\:{M}_{bio}\:$represents the internal state (*µ*) of system, the $\:{fCO}_{2}$ volume fraction the active state (*a*), and the surface temperature *T*_*s*_ (K) the sensory state (*s*) (Fig. 1B). The sensory and active states play the role of boundary or blanket states (*b*), whose trajectories form a Markov blanket between the trajectories of internal and external states^46^(Fig. 1B). The net incoming solar radiation or net insolation $\:{R}_{in}\:$—the incoming minus the reflected solar radiation—became the system’s external states (*η*). In the deterministic model^34,35^, the $\:{R}_{in}$ variable is a so-called “diagnostic variable” and is a deterministic function of $\:{S}_{0}$ and the surface temperature in Eq. (4) (see ODE Eqs. 1–4 in Methods section). In the current stochastic model, we formulate $\:{R}_{in}$ as a stochastic variable that relaxes to the net insolation value, under random fluctuations. Thus, the net insolation $\:{R}_{in}$ defined here may contain stochastic fluctuations such in solar output or surface albedo. So the external state of the system is not the sun itself, but its radiation arriving at the Earth’s surface such that the system could act on. Note that the interactions in the stochastic model are sparser than the sparse coupling required by the FEP (see above), since active states only influence external states via the sensory states. In other words, changes in surface temperature $\:{T}_{s}$ (K) (sensory states) are not functions of the biomass logistic growth $\:{M}_{bio}$ (internal state). Similarly, volume fraction $\:{fCO}_{2}$ (active state) are not functions of $\:{R}_{in}$ (external state) and $\:{M}_{bio}$ and $\:{R}_{in}$ do not influence each other directly, but rather they influence each other via the boundary. This sparse coupling is sufficient to warrant the partition of these four states into external, internal, sensory and active states. This is because the trajectories (i.e. sample paths) of external and internal states are independent when conditioned on sensory and active paths. It is this conditional independence that licences a reading of internal states as encoding Bayesian beliefs over the trajectories of external states. Bayesian beliefs $\:{q}_{\mu\:}\left(\eta\:\right)$ here means strictly that internal states encode a posterior probability density about external states, and active states continuously use this information to regulate sensory states^40,41^.

Insofar as the state partition is established the entailment of active inference at planetary level required a NESS solution in the stochastic model system; a.k.a., random attractor^47^. This randomness means that attracting points or limit cycles of the deterministic dynamics become random attractors that describe the long-term statistical behaviour of the system^48^. Thus, if the stochastic biosphere–climate system has a NESS solution (i.e., a random attractor); at least over the timescale of its existence, it will look as if active states are trying to keep sensory states within their attracting set. Because active states depend upon the internal states one can assert that the latter ($\:{M}_{bio})$ represents and engages active states$\:\:{(fCO}_{2})\:$to maintain sensory states $\:\left({T}_{s}\right)$ within certain bounds. Thus, to determine the NESS solution, we equipped the deterministic model equations of motion of each state variable with random fluctuations, turning the ODEs (Eqs. 1–4) into stochastic differential equations (SDEs, Eqs. 5–8) as shown in methods.

Active inference of external states—by internal states—relies on the existence of a *synchronisation map*, which is a function that maps the most likely internal to the most likely external state, given boundary states. We refer to the most likely internal and external states, given boundary states, as the conditional modes. We denote the synchronisation map (σ) and the internal and external conditional modes as **µ**(b) and **η**(b). The synchronisation function maps the internal to the external conditional mode:

9 $$\sigma \left( {{\boldsymbol{\mu }}\left( {\mathrm{b}} \right)} \right){\mathrm{ }} = {\boldsymbol{\eta }}\left( {\mathrm{b}} \right)$$

Necessary and sufficient conditions for the existence of the function σ have been detailed elsewhere^40,49^. Here, we use a numerical method based on least-squares regression^31^ to identify a synchronisation map of the stochastic biosphere–climate model as a random dynamical system at NESS (see Methods).

## Results

### Stochastic trajectories

**Fig. 2:**
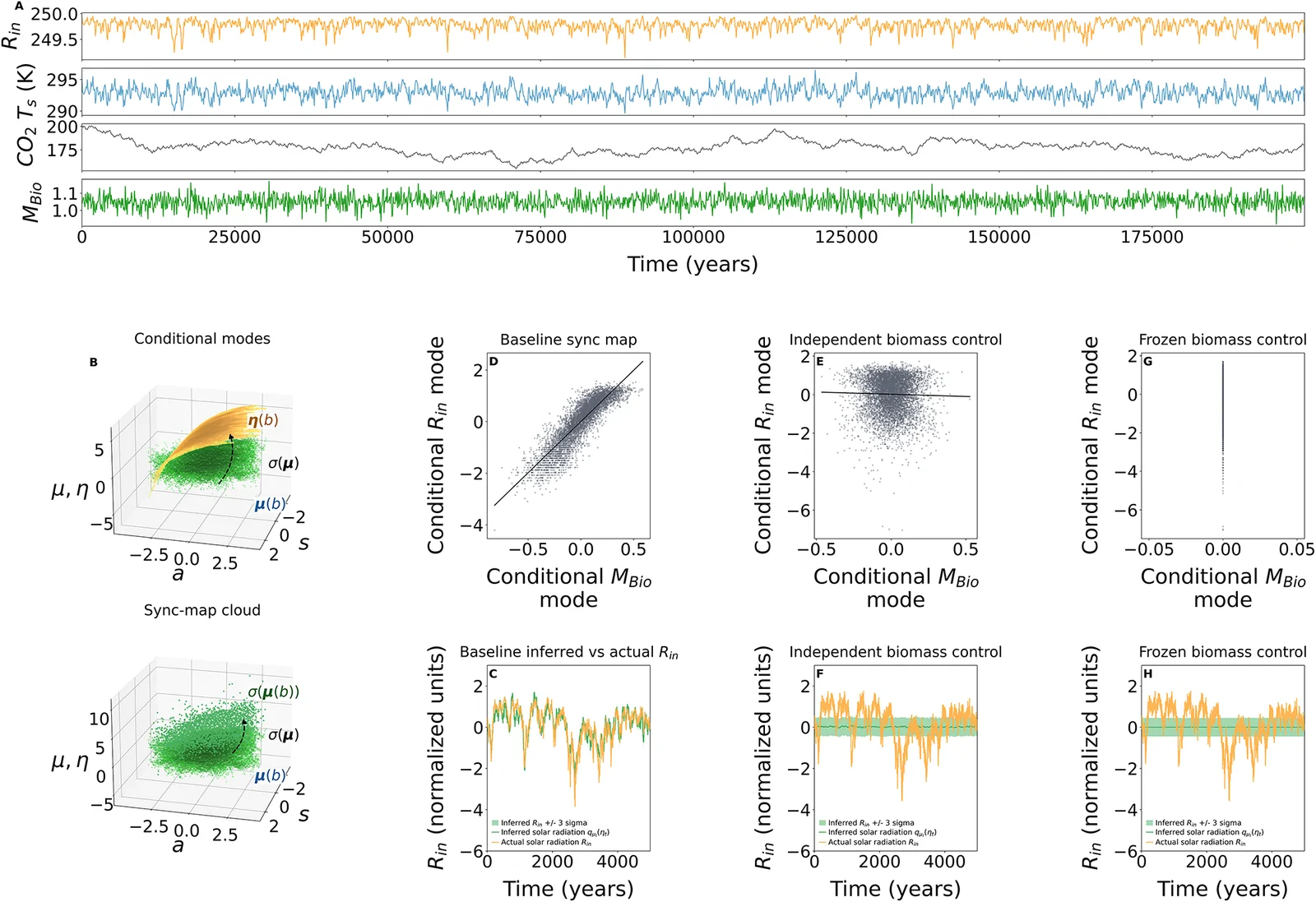
A minimal form of active inference at the planetary scale.** (A)** Representative stochastic trajectory of the baseline nonlinear stochastic biosphere-climate dynamical system model. The $\:{fCO}_{2}$ is a fraction and it is shown in parts per million (ppm) by scaling by $\:{10}^{6}$. The bottom panel shows the 100-year running mean of $\:{M}_{bio}$. **(B)** Boundary-conditional modes and the estimated synchronisation map. The top panel shows the boundary-conditional modes—i.e., the most likely internal states and external states, given boundary states—as a function of the two boundary states b = {* a*,*s*} (x and z axes respectively). Each conditional mode is coloured by its conditional log probability (i.e., $\:lnP\left(\eta\:\right|b)$ or $\:lnP\left(\mu\:\right|b)$). The bottom panel shows the synchronisation map from the (blanket-conditioned) most likely internal state (i.e. the blanket-conditional mode) to the corresponding most likely external state. The predictions of the synchronisation map are shown in green, where the function itself was approximated using linear regression of the most likely external states against the most likely internal states. **(C)** Biosphere-climate’s active inference of shifts in net incoming solar radiation (inferred-vs-actual net incoming-radiation trace). The plot shows the internal states ($\:{M}_{bio}$) of one stochastic sample path of the stochastic dynamical system’s equations of motion (green trace) inferring solar radiation $\:{R}_{in}$ given blanket states ($\:{T}_{s}$ and $\:{fCO}_{2}$) as a result of applying the estimated synchronisation map to the trajectory of internal states $\:{M}_{bio}$. The prediction of external *variances* is shown (light green) as a confidence interval around the predicted mode (dark green). The yellow trace is the concurrent sample path of the external states (solar radiation) from the same stochastic realisation, to illustrate the precision of the internal states tracking of the external states, via the synchronisation map. **(D)** Baseline conditional-mode synchronisation map of C. **E-F)** Independent-biomass control, in which biomass remains stochastic but is dynamically decoupled from the carbon-cycle weathering and burial terms. **G-H)** Frozen-biomass control, in which biomass is held constant and removed from the carbon-cycle weathering and burial terms.

For all numerical analyses we simulated *N* = 100 independent stochastic realisations of the biosphere-climate dynamical system for T = 200,000 years. Figure 2A shows an example of one such stochastic realisation of the system’s equations of motion with an additional 100-year moving average of the biomass variable $\:{M}_{bio}\:$ (Fig. 2A, bottom subplot). To exclude the effects of transients—before the attainment of NESS—all analyses were conducted after an initial ‘burn-in’ period of $\:{10}^{5}$ years, at which point steady-state was assumed to be attained. The following results are based on the final $\:{10}^{5}$ years of each realisation. Large fluctuations in $\:C{O}_{2}$ are driven by stochastic forcing, but the mean state and the restoring drift toward its homeorhetic trajectory are shaped by the biomass-dependent carbon-cycle terms (Supplementary Fig. 1). This stabilizing effect of biomass to influence the mean $\:C{O}_{2}$ supports the synchronisation between internal and external variables.

### Synchronisation map

In this set of analyses, we use regression analysis to estimate the boundary-conditional modes (left panel) and the synchronisation map ($\:{\sigma\:}_{est}$) (right panel) (Fig. 2B) (see Methods). The boundary-conditional modes are shown as an orange point-cloud of the most likely internal states **µ**(b) and most likely external states **η**(b) (Fig. 2B left panel), while the estimates of the synchronisation map are shown as an green point-cloud that maps the (boundary-conditioned) most likely internal state **µ**(b) (i.e. the boundary-conditional mode) to the parameter of a posterior belief about the most likely external state, denoted $\:{q}_{\mu\:}\left(\eta\:\right)$ (Fig. 2B right panel).

Both panels are plotted as a function of the two boundary states b = {* a*, *s*} (x and z axes, respectively). Each point is coloured according to the log-probability the mode is assigned under its respective conditional distribution. The more accurate the estimated synchronisation map is, the more closely the green point-cloud (predicted external modes) in the right panel should overlap with the orange point-cloud (actual external modes) in the left panel. Once determined, the ensuing conditional synchronisation map can be used to map internal states ($\:{M}_{bio}$) to an approximate posterior expectation about the most likely value of the external states ($\:{R}_{in}$). That is, the green point-cloud can be seen as a prediction of the orange point-cloud.

### Active inference

Applying an estimated synchronisation map $\:{\sigma\:}_{est}\:$to a trajectory of internal states ($\:{\mu\:,\:M}_{bio}$), allowed us to imbue the dynamics of the biosphere-climate system with a Bayesian posterior belief (see Eq. 11) over the most likely value of external states ($\:\eta\:$, $\:{R}_{in}$), given the current value of the sensory states (surface temperature, $\:{T}_{s}$) and active states (volume fraction of $\:fC{O}_{2}$). Figure 2C shows the internal states tracking the most-likely value of external states. The inference appears not only to be accurate on average, but in fact internal states appear to momentarily track the sample paths of external states, either when there is sudden increase or decrease in $\:{R}_{in}$. The paths of internal states ($\:{M}_{bio}$) of the stochastic biosphere-climate system can thus be described as encoding a Bayesian posterior belief over most-likely value of external states, parameterized with a conditional mean $\:\sigma\:\left(\mu\:\right)\:$and a conditional variance $\:\varsigma\:\left(\mu\:\right)\:$that allows prediction of external *variances*, shown as a confidence interval (light green) around the predicted mode (dark green) (Fig. 2C).

We have run a falsification control experiment to test the role of biosphere in the system’s inference by altering the differential equations of the biosphere-climate system model. When the biosphere ($\:{M}_{bio}$) is extinct (frozen) from the climate system model or decoupled from active states (by eliminating the biomass-enhanced weathering and biomass-dependent burial terms) the synchronisation map (Fig. 2E, G) and the active inference of the system fails (Fig. 2F, H), whereas the baseline model retained a non-trivial synchronisation (Fig. 2D) and active inference signature (Fig. 2C). Both controls disrupt the baseline synchronisation and tracking relationship, showing that active inference depends on biologically mediated coupling to the climate-carbon dynamics pointing the importance of active states (*a*).

### Sensory perturbation

**Fig. 3:**
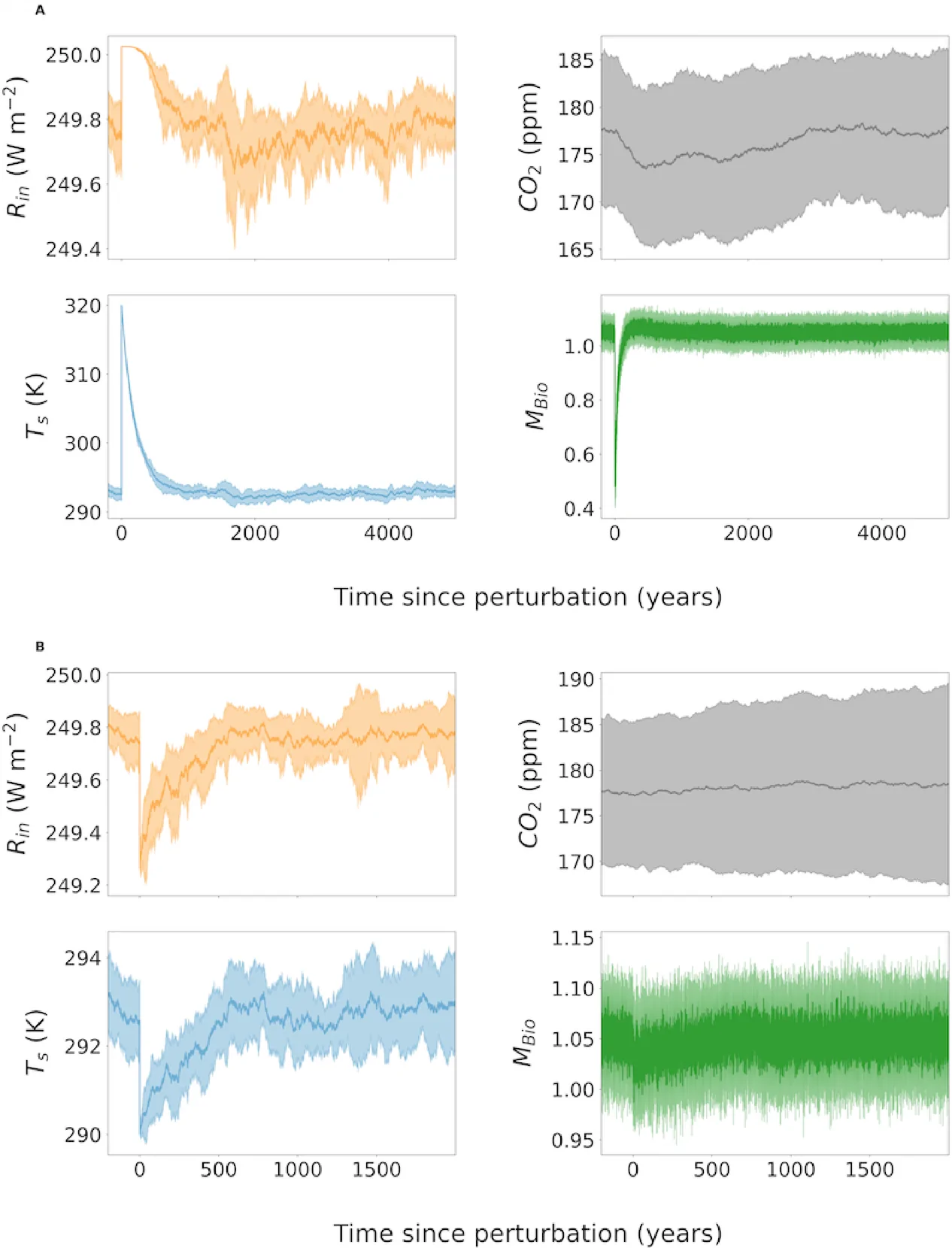
Sensory perturbation and climate homeorhesis. The plot shows the response of the system to a fixed perturbation to the sensory state $\:{T}_{s}$ (blue), where $\:{T}_{s}$ at a particular time-point was artificially clamped to a value of 320 K (**A**) and 290 K (**B**) and then allowed to relax for 5000 years (50,000 timesteps) (**A**) and 2000 years (20,000 timesteps) (**B**). Lines show ensemble means and shaded regions show standard deviation across trajectories. Average responses with standard deviation across *N* = 100 parallel realisations are shown. In all panels the perturbation is introduced at time t = 0. The biosphere-climate system’s dynamics are shown for a few hundred years before each perturbation to illustrate the effect of the perturbation in pushing the system away from its baseline, unperturbed homeorhetic dynamics.

In the next set of analyses, we analysed the predictive, active inference interpretation of the biosphere-climate system by subjecting the sensory states (surface temperature $\:{T}_{s}$) to perturbations. Figure 3 shows the results of these perturbation experiments, summarised across *N* = 100 independent realisations. For each perturbation, we clamp the surface temperature $\:{T}_{s}$ to a fixed temperature, either 320 K (Fig. 3A) or to 290 K (Fig. 3C), and then let the dynamics transiently relax for 2000 years.

When the surface temperature is perturbed, internal states ($\:{M}_{bio}$) rapidly respond by changing active states ($\:{fCO}_{2}$). When the sensory state (surface temperature $\:{T}_{s}$) is clamped to 320 K, the internal states $\:{M}_{bio}$ respond rapidly (around 250 years) to the disturbance, with a negative deflection (Fig. 3A). This rapid response of internal states affects the active states by decreasing the $\:{CO}_{2}$ volume fraction, hence the greenhouse effect. Notice that when this happens, the volume fraction of $\:{CO}_{2}$ and the surface temperature $\:{T}_{s}$ return to their homeorhetic trajectory prior to disturbance, although the $\:{CO}_{2}$ takes longer (more than 2500 years) than the $\:{T}_{s}$ (Fig. 3A, Suppl. Figure 2). When the sensory state $\:{T}_{s}$ is perturbed to 290 K, there is a slow positive deflection of internal states $\:{M}_{bio}$ that takes around 500 years to reach the steady state prior to perturbation (Fig. 3B). A slight increase of $\:{CO}_{2}$ that does not return to the volume fraction prior to disturbance is also observed. In both cases (disturbance to 320 K or to 290 K) the surface temperature $\:{T}_{s}$ returns to the same values of the steady state prior to disturbance, although when the temperature is clamped at 290 K, the return to steady state is faster (around 500 years) and the variation is greater compared to the perturbation of 320 K (around 750 years) (Fig. 3A, B).

In both cases changes in the sensory states (surface temperature $\:{T}_{s}$) involve changes in the dynamics of active states (*f*$\:{CO}_{2}$), while the response of the internal states is faster when the $\:{T}_{s}$ is increased to 320 K (Fig. 3A, B). The changes in active states are of greater magnitude when the surface temperature is increased, and the variation of internal states is of greater magnitude when the surface temperature is decreased. Through the active and sensory states, the dynamics of the biosphere-climate system acts on the external states ($\:{R}_{in}$), such that the posterior belief of these being outdated returns to their steady state, although it takes longer when $\:{T}_{s}$ is increased to 320 K (Fig. 3A).

### Parameter modification

**Fig. 4:**
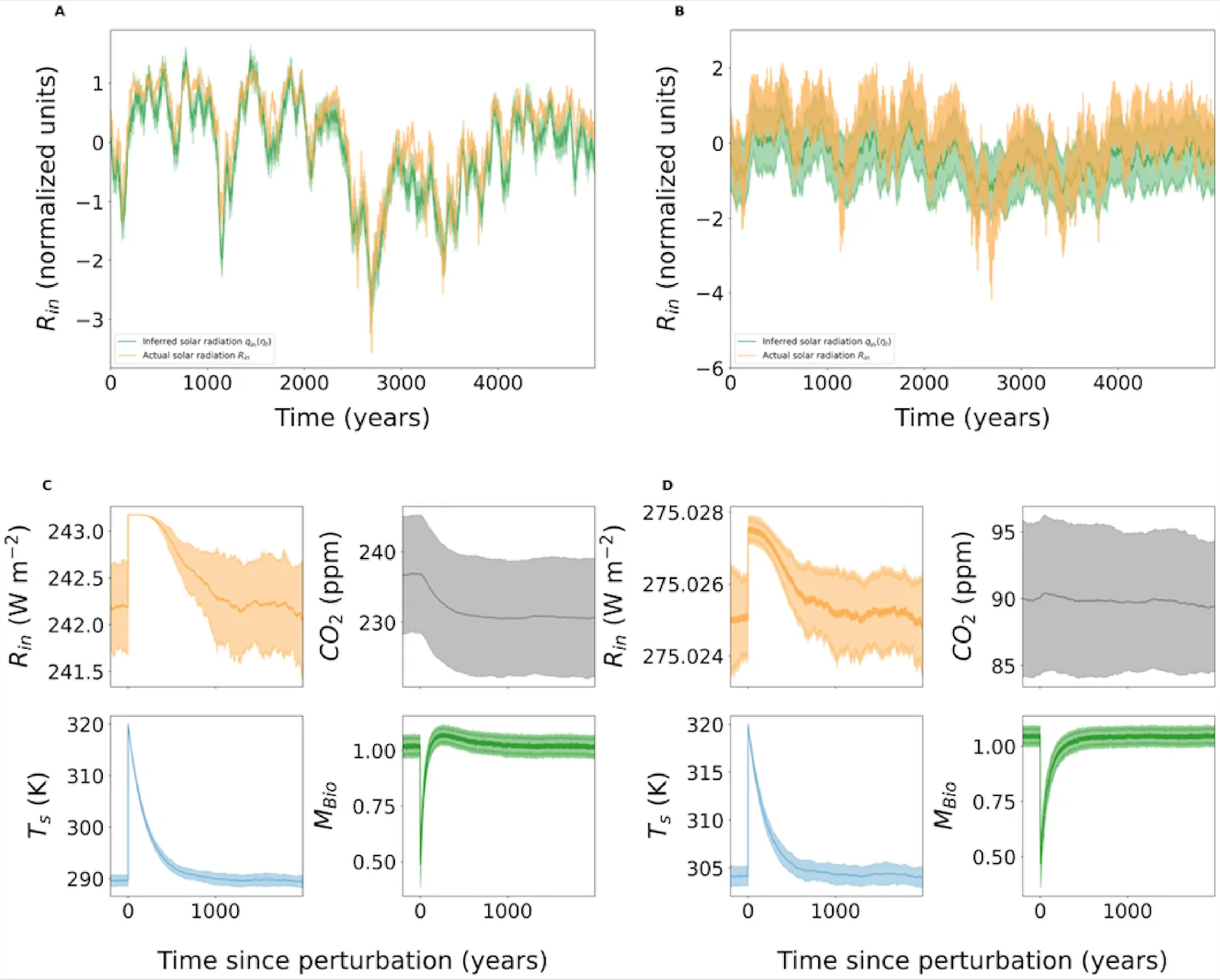
Response to acute parameter change.** (A)** The plot shows the active inference of the system when changing albedo ($\:{\alpha\:}_{1}$) parameter from 0.43 to 0.45 and **(B)** changing arbitrarily solar constant $\:{S}_{0}$ parameter from 1370 $\:W{m}^{-2}$ to 1507 $\:W{m}^{-2}$. **C**,** D)** Sensory perturbation under parameter change $\:{\alpha\:}_{1}$ (C) and $\:{S}_{0}$ (D).

In these analyses, we asked whether the effectiveness of climate homeorhesis is affected by the system parameters that relate to energy balance—such as albedo $\:{{\upalpha\:}}_{1}\:$and solar constant $\:{\mathrm{S}}_{0}$ of active–boundary–states. We were interested to know how specific values of the albedo $\:{\alpha\:}_{1}\:$and $\:{S}_{0}$ influenced the precision of the inferential process when the system is perturbed and unperturbed (Fig. 4).

Changing the albedo $\:\left({\alpha\:}_{1}\right)\:$parameter does not abolish the active inference of the biosphere-climate system tracking the net incoming solar radiation (Fig. 4A). However, when the solar constant $\:\left({S}_{0}\right)$ parameter is modified from the past observed value 1370 $\:W{m}^{-2}$ (for which the deterministic biosphere-climate systems model has been built)^34,35^ to an arbitrarily (not realistic in the near future) value of S_0_ = 1507 $\:W{m}^{-2}$, the precision of active inference is weakened but stable, and its average variation increases with respect to the fluctuation of the external states ($\:{R}_{in}$) (Fig. 4B). In general, when compared with the original parameters of the model (Fig. 2C), the precision of the implicit active inference remains similar when $\:{\alpha\:}_{1}$is modified, while in $\:{S}_{0}$ tends to decrease (Fig. 4A, B).

Sensitivity analysis of parameter change shows that when the surface temperature $\:{T}_{s}$ of the system is clamped to 320 K and the dynamics relax, in the case of modified $\:{\alpha\:}_{1}$ parameter ; the sensory states $\:\left(s\right)\:$returns smoothly to its prior trajectory and thus of climate homeorhesis (steady-state) after around 2000 years post-perturbation (Fig. 4C). When a similar disturbance is imposed while the $\:{S}_{0}\:$parameter is modified (Fig. 4D); surface temperature $\:{T}_{s}$ (sensory states), return to its trajectory prior to the disturbance, yet slower, erratically, and with greater variation than in the $\:{\alpha\:}_{1}$ modification (Fig. 4D). The response of internal states $\:{M}_{bio}$ is also slower when compared to modified $\:{\alpha\:}_{1}$ and active states *f*$\:{CO}_{2}$ remain with similar dynamics prior to perturbation.

Additional sensitivity analyses were performed by changing the solar constant less abruptly, from S_0_ = 1370 $\:W{m}^{-2}\:$to current updated consensual value S_0_ = 1360 $\:W{m}^{-2}\:$^51^, while keeping the rest of the model and analysis pipeline unchanged. The inference and synchronisation results are not weakened in non-arbitrary modification (Suppl. Figure 3). Active inference remains robust, just as in the unmodified model (Fig. 2C).

### Robustness analysis

In this final set of analysis, we focus on a robustness analysis of the synchronisation map as a function of the solar constant $\:{S}_{0}$, and on to test the robustness of the active inference result as a function of model variations.

Because measuring the synchronisation map’s robustness is effectively measuring the “viable range” of $\:{S}_{0}$ this means that we have identified the $\:{S}_{0}$ parameter limit values ($\:{S}_{0}$ < lower threshold, $\:{S}_{0}$ > upper threshold) over which active Bayesian inference is robust (i.e., precise), and therefore how well internal states ($\:{M}_{bio}$) track external states ($\:{R}_{in}$), using the Pearson’s Rank correlation^51^ between inferred external trajectories $\:{q}_{\mu\:}$(η), and actual external trajectories $\:{R}_{in}$.

**Fig. 5:**
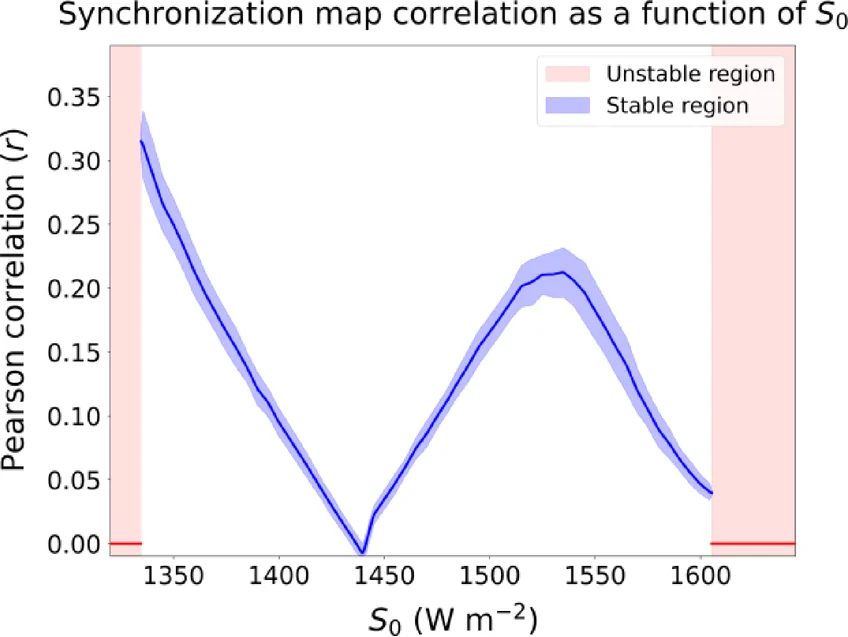
Robustness analysis. This plot quantifies the synchronisation map in terms of the correlation coefficient (averaged across *N* = 1 realisations) between the posterior belief parameterized by the internal states $\:q(\eta\:;\:{\sigma\:}_{\mu\:}(\mu\:),\:{\sigma\:}_{\varSigma\:}(\mu\:\left)\right)$ and the simultaneous external states $\:{\eta\:}_{t}$, as a function of the solar constant ($\:{S}_{0}$).

This analysis showed that the accuracy of external estimates afforded by the synchronisation map has a nontrivial relationship with the solar constant $\:{S}_{0}$: inference is robust within some lower and upper $\:{S}_{0}$ parameter limit values. When $\:{S}_{0}$ decreases from its default value (1370.0 W$\:{m}^{-2}$) the Pearson’s Rank correlation increases up to 1340 W$\:{m}^{-2}$, while it appears to change non-monotonically as $\:{S}_{0}$ increases to 1610 W$\:{m}^{-2}$ (Fig. 5). In other words, within this $\:{S}_{0}$ parameter range, the correlations change non-monotonically. Below than $\:{S}_{0}$*=*1340 W$\:{m}^{-2}$ and above 1610 W$\:{m}^{-2}$ the synchronisation, and as a consequence the synchronisation map itself becomes undefined. We classified the system as unstable when the numerical solution for the realisations did not reach a steady-state (indicated computationally through numerical over- or under-flow), indicating vanishing or exploding dynamics, or a very long transient time.

To test the robustness of the active inference result as a function of model variations we have altered the biosphere-climate’s differential equations, both simplified on the one hand, and augmented with extra degrees of freedom on the other.

In the first case a set of simplifications focused specifically on the atmospheric $\:{CO}_{2}$ Eq. 7 (see methods). None of these simplifications improved the active inference and synchronisation results (Suppl. Figure 4). The baseline equation gave the strongest performance, with mean inferred-vs-actual $\:{R}_{in}$ correlation across trajectories of 0.882. Increasing the weathering exponent weakened the result slightly (*n* = 0.7: mean correlation 0.868; *n* = 1.0: mean correlation 0.860). Removing the biomass-dependent burial sink weakened the result further (mean correlation 0.835) (Suppl. Figure 4). The strongest degradation among the $\:{CO}_{2}$-equation simplifications occurred when the biomass enhancement of silicate weathering was removed: Ω = 0 reduced the mean correlation to 0.682 and reversed the fitted sync-map slope. Removing both biomass-dependent $\:{CO}_{2}$ sink terms (Ω, $\:{\beta\:}_{NNP})$ also degraded the result substantially (mean correlation 0.742; sync-map slope = -4.29) (Suppl. Figure 4). This case is important because it can still produce a mathematical synchronisation map in conditional-mode space, but the sign reversal and degraded prediction mean that we do not interpret it as preserving the same active inference relation. The independent-biomass / decoupled-carbon-cycle control gives the strongest falsification result, with mean correlation near zero (0.001) and sync-map slope − 0.23.

In the second case we examined an augmented model in which the original single biomass state is replaced by two biomass variables with different optimal temperatures, while the rest of model variables remain shared. The synchronisation-map result in this two-$\:{M}_{bio}$ “5D” (5-dimesional model) is strong using the raw conditional-mode readout. Using blanket-conditioned conditional modes with a mode-based estimator, the fitted sync-map gives R2 = 0.819 and the representative trajectory gives an inferred-vs-actual $\:{R}_{in}$ correlation of 0.951 (Suppl. Figure 5). We further analysed a “7D” stochastic biosphere-climate model with additional degrees of freedom (two temperature, two biomass, two solar radiation) with separate high- and low-latitude temperature, biomass, and net incoming-radiation variables, coupled through a shared atmospheric $\:{CO}_{2}$ state (Suppl. Figure 6). In this 7-dimensional version, the inference synchronisation result is still present, but it is weaker than in the 4 and 5-variable model, yet still demonstrable.

Finally, we performed a focused sensitivity analysis over the optimal biomass temperatures in the 7D model, varying $\:{T}_{bio\_high}$ over 8, 12, and 16 °C and $\:{T}_{bio\_low}$ over 22, 26, 28, 30, and 34 °C. This was motivated by the weak low-latitude $\:{R}_{in}$ result in the default 7D parameterisation. Lowering the low-latitude optimum from 28 to 22 °C improved the low-latitude inferred-vs-actual correlation modestly (Suppl. Figure 7). In the most balanced full rerun, $\:{T}_{bio\_high}$ = 16 °C and $\:{T}_{bio\_low}$ = 22 °C high-latitude correlation 0.412, low-latitude correlation 0.148, and multivariate sync-map R2 = 0.832. The result indicates that the weaker low-latitude tracking in the 7D model is parameter-sensitive, but still weaker than the baseline four-variable (Fig. 2C). Thus, the weak low-latitude result is somewhat parameter-sensitive.

## Discussion

This study aligns somehow with the hypothesis of the *“biosphere as an active adaptive control system capable of maintaining the Earth in homeostasis*”^13^ and thus the idea that there is a form of single coupled biosphere-climate system operating at a global level that maintains temperature and chemistry among other factors at habitable states for whatever is the current biota^15^. Our formal treatment substantiates this claim by showing that Earth’s climate non-equilibrium steady state dynamics—that underwrite habitability—entails active inference operating at global level.

The active inference formulation of the biosphere–climate system developed here enables us to interpret the Earth system as exhibiting a possible and plausible elementary form of agency—the capacity to act autonomously to maintain geophysiological bounds. A connection between Daisyworld and a form of reactive perception has been explored elsewhere^52,53^, although without incorporating Earth-specific biophysical parameters such as albedo or the solar constant, nor any active predictive behaviour, making it less comparable to our approach. Different approaches to associative network learning at the ecological level^54,55^ seems to be analogous or overlap with active inference. Indeed, the free energy principle can integrate models of associative learning proposed from empirical experiments and serve as a framework for a better understanding systems behaviour^56^.

Recent approaches to the Gaia hypothesis suggest that climate homeorhesis can emerge by a process of ‘sequential selection’ across multiple scales through ‘selection by survival alone^57^. The fact that the system search for a stable attractor is similar and complementary to what is presented here. Active inference as sequential selection can be read as a new take on good regulator theorem and ultrastability^58,59^. Whereas sequential selection is a bottom-up, active inference is a top-down approach to the Gaia phenomenon^29^.

The mechanisms behind active inference at planetary scale, in biosphere-climate system terms, occur through the coupling of biomass, atmospheric $\:{CO}_{2}$, and surface temperature in reference to solar forcing fluctuation. Biomass responds to climatic conditions while simultaneously modifying them through carbon uptake and sequestration. Variations in solar forcing and stochastic perturbations function as external signals that trigger changes in temperature and atmospheric $\:{CO}_{2}$ concentrations. Through biomass-dependent carbon-cycle processes, the biosphere reduces deviations from favorable environmental conditions by increasing $\:{CO}_{2}$ uptake when concentrations rise and declining when conditions become unfavorable. This creates a restoring loop that maintains temperature and carbon levels within habitable ranges. This happens until the Sun’s evolution brightness becomes untenable.

In active-inference terms, the biosphere–climate system—endowed with boundary (sensory and active) states—maintains its surface temperatures in homeorhesis by implicitly inferring the sensory causes of external perturbation. That is, the system continuously minimizes discrepancies between current external states and those compatible with sustained internal states dynamics. The system can be read as inferring sensory causes of net insolation changes, acting accordingly by changing its boundary states and thereby engaging in predictive behaviour, whence the states of the biosphere encode Bayesian posterior probability density^40,41^ over external causes of sensory perturbation (shifts in net incoming solar radiation). Moreover, this posterior probability density is actively updated through changes in the volume fraction of *CO*_*2*_ and in the face of new signals (information) sensed through the surface temperature *T*_*s*_, that is, through the system’s active and sensory states respectively. The resulting synchronisation between internal ($\:{M}_{bio}$), active (atmospheric *CO*_*2*_), and sensory (*T*_*s*_) states can therefore be interpreted as a primitive form of planetary active inference operating through active states (carbon-cycle) regulation. The system’s inference seems not only to track external states on average, but also seems to track every realisation or sample path. This seems to validate the identification of active and sensory states in the system, supporting a previous proposal about how the Earth system may look like and potentially behave with a Markov blanket-like boundary^32^. It is justified in the way that the Earth’s atmosphere has been self-produced, modified and modulated by the biosphere through the geological eons^21,60^ and the implication it has for surface temperature, and hence climate dynamics.

Given the vast changes in plate tectonics regime and weathering regimes over the entire life span of Earth, whether the estimations of the system inference, sampled in a millennial time-scale, could be generalised to geological time scales is an open question. In theory, is not necessary to sample a longer time series or data over very long timescales to perform the Bayesian analysis of the Earth system as engaging in active inference. This is because the correspondence between the boundary-conditional modes—via the estimated synchronisation map—suggests an accurate approximation to the NESS density around the mode, and that the system explores its state space straightforwardly, so that this mode is visited frequently. This means that the synchronisation map can be characterised even from relatively short trajectories, given the dynamics spend sufficient time nearby the conditional modes.

The results of increasing the degrees of freedom in both the 5 and 7 dimension variants of the model shows that active inference is not limited or an artefact of the low 4-dimensionality of the baseline biosphere-climate model. Under these modifications, the system still preserves active inference behaviour and the main conditional internal-external states relationship. Active inference remains robust under a biologically augmented two-biomass formulation (5-dimentional model), which emulates (in an albeit simplistic manner) the different biospheres that the Earth has had. Nevertheless, even the more complicated biosphere–climate system model still has several limitations. Several key complex processes—such as cloud dynamics, ocean circulation, trophic interactions, nonlinear transitions at multiple scales, which are important for Earth system dynamics—are not parameterized, which limits the generalisability and extrapolation of the results.

The biosphere-climate system model is best understood here as a heuristic and exploratory tool: it effectively illustrates how a biosphere–climate system can implement active inference. Future analyses should consider more comprehensive models of the Earth system, while the way in which the system is partitioned is also key. That is, it depends on the particular way the $\:{M}_{bio}$ and $\:{R}_{in}$ variables are coupled to each other. We have followed a previous line of reasoning that we believe is consistent with geomicrobiological evolution, which should not be overlooked^32^, and here we provide a proof of concept, based on that line of reasoning about the possibility of inferential dynamics at planetary scale. In other words, the plausibility that the biosphere–climate system encodes a posterior probability density (Bayesian beliefs) about its—solar—environment, and that it behaves according to those beliefs. This interpretation goes beyond simple synchronisation because the model estimates not only a conditional mean for the external state, but also a conditional variance, allowing the internal states to parameterize to approximate posterior density over net incoming radiation. Importantly, the external state in the model is not solar luminosity itself, but net incoming radiation at the Earth’s surface, after reflection by albedo (incoming minus outgoing solar radiation). The simplification tests further support this interpretation: when biomass is either frozen or dynamically decoupled from the climate–carbon system, the active inference and synchronisation tracking signatures are lost. This indicates that the result is not a generic artefact, but depends on biologically mediated coupling within the climate–carbon dynamics and the well identification of the Markov-blanket partition.

By describing Earth’s biosphere–climate dynamics in terms of active inference, our results support the theory that geophysiological functioning is an integrated process of the entire Earth system, rather than of the biosphere alone^61^. Indeed, the sensory perturbation we imposed on the system results in a highly integrated response, under which sensory dynamics are dependent on biosphere (internal) and *CO*_*2*_ (active) states to keep surface temperature within an attracting set (i.e., random attractor). When we clamped the temperature of the sensory states below its optimal parametric set up, the internal states of the system resist such perturbation, through active states, i.e. by increasing *CO*_*2*_ volume fraction, hence greenhouse effect. The contrary is also valid. When sensory states are held above their characteristic value, there is a reduction of *CO*_*2*_ volume fraction and the overall dynamics of the system remain within geophysiological bounds. Such results connect with recent data-driven analyses that show long-term NESS climate dynamics over geological timescales despite large external perturbations^62^ and statistical evidence that supports the observation of homeorhetic-like properties at the planetary-climatic scale^63,64^.

Modifying system parameters that relate to energy balance—such as albedo $\:{{\upalpha\:}}_{1}\:$and solar constant $\:{\mathrm{S}}_{0}$—changes the properties of the synchronisation map and therefore affects the precision of inference. The inference becomes less accurate when the albedo parameter is modified from 0.43 to 0.45, within the realistic standard values between the α_*ice*_ = 0.62 and that of a fully deglaciated planet, α_*free*_ = 0.30^65^. Similarly, when solar constant $\:{\mathrm{S}}_{0}$ parameter is changed from 1370 $\:W{m}^{-2}$ to 1507 $\:W{m}^{-2}$ the inference changes quantitatively. Outside of this window, numerical analyses suggest that there is no NESS.

Parametric values influence the sensory states of the system and thus the synchronisation map, which determines how well the internal states ($\:{M}_{bio}$) can instantaneously infer the most likely value of external states ($\:{R}_{in}$). This means that these parameters, at least in the analysed time window, underwrite the synchronisation between internal and external states and hence for NESS. In general, a well-fitted synchronisation map is a necessary condition that reflects a high mutual information across the system; in the sense that the system can infer sensory causes of sun radiation at the Earth’s surface, change active states (*fCO*_*2*_ volume fraction), which are driven by biosphere dynamics, and thus regulate sensory states (climate temperature dynamics).

The Pearson correlation analyses show that when solar luminosity remains between 1310 $\:W{m}^{-2}$ and 1600 $\:W{m}^{-2\:}$the synchronisation map is stable; the internal states are non-trivially correlated with the external states. This corresponds to the viability ranges over which inference is robust in the sense that there is a NESS and the internal sates are non-trivially correlated with the external states. Roughly speaking, this variability in the correlation between these values corresponds to the same period of the transition from an early microbial anaerobic biosphere^66,67^ to the future lifespan of the biosphere, ≈ 1.5 billion years beyond the present^8–10^, whence the solar constant will reach at least 1576 $\:W{m}^{-2}$^3^. For values of solar constant outside of 1310 $\:W{m}^{-2}$ and 1600 $\:W{m}^{-2\:}$the inference is no longer tenable, in the sense that no NESS solution exists within this region of parameter space. Nevertheless, there is a need to study this more comprehensively in future work.

Predictive behaviours are “cost-effective”, because acting such so as to avoid a predicted deviation/perturbation (anticipation) is generally much cheaper than correcting a deviation (feedback). It is therefore not surprising to find empirically such behaviours at different levels of biological organisation, from microbes^68^, parasites^69^, myxomycetes^70^ and the trees of the Amazon rainforest^71^— a puzzling feature so far in the context of the whole biosphere-climate system. The analyses shown here present a heuristic proof which suggests that similar behaviour could happen on a planetary scale.

## Methods

### Deterministic biosphere-climate dynamical system model

The deterministic dynamical system model studies the long-term dynamical evolution of the interaction between the biosphere and climate^34,35^. The deterministic model’s state space comprises the Earth’s surface temperature $\:{T}_{s}$ (K), the Biomass $\:{M}_{bio}$, and the volume fraction $\:{fCO}_{2}$ as follows: 

1 $$c\frac{{dT_{S} }}{{dt}} = R_{{in}} - A_{0} - B_{0} (T_{S} - 273.15) + A_{\mathrm{CO}_2}\ln\left[ {\frac{{fCO_{2} }}{{fCO_{2}^{0} }}} \right]$$

2 $$\frac{{dM_{{bio}} }}{{dt}} = \left[ {\left( {M_{{\max }} - M_{{bio}} } \right)U_{0} \exp \left( { - \frac{{\left( {T{}_{s} - T_{{bio}} } \right)^{2} }}{{2\sigma _{{bio}}^{2} }}} \right) - 1} \right]M_{{bio}}$$

3 $$\mu_{Atm} \frac{{dfCO_{2} }}{{dt}} = V_{0} - W_{o} \exp \left( {\frac{{T{}_{s} - T_{{sil}} }}{{\Delta T_{{sil}} }}} \right)\left[ {\frac{{fCO_{2} }}{{fCO_{2}^{0} }}} \right]^{n} \left( {1 + \Omega M_{{bio}} } \right) - \beta_{NPP} M_{{bio}}$$

4 $$R_{{in}} = \frac{{rSo}}{4}\left( {1 - \alpha _{1} + \alpha _{2} \tanh \left[ {\frac{{T{}_{s} - T_{{ice}} }}{{\Delta T_{{ice}} }}} \pi\right] } \right)$$

where $\:{S}_{0}$ is the solar constant with a reference value $\:{S}_{0}$ = 1370 W$\:{m}^{-2}$. $\:{R}_{in}$ is the net incoming shortwave radiation at the Earth’s surface. In this deterministic model, there is a 3-way dynamical system and a diagnostic variable $\:{R}_{in}$ that captures the nonlinear relationship between the $\:{T}_{s}$ and $\:{R}_{in}$, which couples back to Eq. 1.

The parameter values are given in Table 1. Some parameters are given in the unit of *seconds* and others are in the unit of *years*. Thus, the parameters with the unit *1/s* is scaled to those of the unit *1/yr* by multiplying the factor $\:\tau\:\:$=31,557,600 = (365.25 × 24 × 60 × 60) s/yr. Then we divide Eq. (1) by the heat capacity c = 1.20 x$\:{\:10}^{10}{JK}^{-1}{m}^{-2}$ and Eq. (3) by the present atmospheric load $\:{\mu\:}_{Atm}$ =1.77 x $\:{\:10}^{20}$ mol. The parameters obtained after the scaling and/or the division are shown with tilde $\:\stackrel{\sim}\cdot$ (e.g., $\:{\stackrel{\sim}{A}}_{0}$= ($\:\tau\:\:$/c)$\:{A}_{0}$ [K/yr]).

### Stochastic biosphere-climate dynamical system model

To convert the ordinary differential equations (ODEs) above into a random dynamical system, we convert the ODEs, Eqs. (1)-(4), to stochastic differential equations (SDEs) by adding random fluctuations to the deterministic equations of motion of each state variable as shown in the following Eqs. 5–8.

5 $$\dot{s}:\frac{{dT_{S} }}{{dt}} = (\tau /c)R_{{in}} - \tilde{A}_{0} - \tilde{B}_{0} (T_{S} - 273.15) + \tilde{A}_{{CO_{2} }} \ln\left[ {\frac{{fCO_{2} }}{{fCO_{2}^{0} }}} \right] + \omega _{1} \xi _{1} (t)$$

6 $$\dot{\mu }:\frac{{dM_{{bio}} }}{{dt}} = \left[ {\left( {M_{{\max }} - M_{{bio}} } \right)U_{0} \exp \left( { - \frac{{\left( {T_{s} - T_{{bio}} } \right)^{2} }}{{2\sigma _{{bio}}^{2} }}} \right) - 1} \right]M_{{bio}} + \omega _{2} \xi _{2} (t)$$

7 $$\dot{a}: \frac{{dfCO_{2} }}{{dt}} = \tilde{V}_{0} - \tilde{W}_{o} \exp \left( {\frac{{T_{s} - T_{{sil}} }}{{\Delta T_{{sil}} }}} \right)\left[ {\frac{{fCO_{2} }}{{fCO_{2}^{0} }}} \right]^{n} \left( {1 + \Omega M_{{bio}} } \right) - \beta_{NPP} M_{{bio}} + \omega _{3} \xi _{3} (t)$$

8 $$\dot{\eta }:R_{{in}} = \gamma \left\{ {\frac{{So}}{4}\left( {1 - \alpha _{1} + \alpha _{2} \tanh \left[ {\frac{{T_{s} - T_{{ice}} }}{{\Delta T_{{ice}} }}} \pi\right] } \right) - R_{{in}} } \right\} + \omega _{4} \xi _{4} (t)$$

Furthermore, we assume $\:{R}_{in}$ that is a dynamical variable that relaxes quickly to the state given by Eq. (4), and assume a large value for γ (γ = 10) so that the effect of the solar constant is controlled solely by $\:{R}_{in}$. Consequently, $\:{R}_{in}$ is transformed into its own prognostic variable in Eq. (8). We are taking the influence of the temperature on itself and extracting a new variable, which represents the incoming minus the reflected radiation. To satisfy the mathematical prerequisite of independent dynamical variables for the active inference blanket, this formulation introduces this relaxation time and noise into $\:{R}_{in}$. While this approach serves as a mathematical expediency for a proof of concept rather than a strict replication of instantaneous physical radiative processes, fully exploring the limit where this delay vanishes (γ$\:\to\:\infty\:$) falls outside the scope of this study.

In the simulation we use γ = 10 (1/yr), $\:{\omega\:}_{1}$ = 0.1, $\:{\omega\:}_{2}$= 0.1, $\:{\omega\:}_{3}$ = $\:{10}^{-7}$, $\:{\omega\:}_{4}$ = $\:{10}^{-3}$, and the other parameter values in both partitions are taken from Table 1 except for $\:{\alpha\:}_{1}$. We modify the first albedo parameter from $\:{\alpha\:}_{1}$= 0.46 to $\:{\alpha\:}_{1}$ = 0.43.

For all analyses, we simulated *N* = 100 independent sample paths of the stochastic biosphere-climate system by integrating the stochastic process for 2x$\:{10}^{5}$ years with a step size of 0.1 years, using a standard Euler-Maruyama integration scheme^72^. The initial condition used was $\:{T}_{s}$(0) = 295 K, $\:{M}_{bio}$(0) = 1, $\:{fCO}_{2}$ = 2x$\:{10}^{-4}$, and $\:{R}_{in}$(0) is the value given by Eq. (4). The atmospheric $\:{CO}_{2}$ mixing ratio was taken to be governed by a simple long-term carbon cycle model. The organic carbon burial rate is assumed to increase linearly with biomass. The silicate-weathering rate has been assumed less temperature dependent, and the volcanism as constant. There is no partitioning of the $\:{CO}_{2}$ reservoirs between ocean and atmosphere.

In the model, $\:{fCO}_{2}$ denotes the atmospheric mixing ratio or volume fraction of $\:{CO}_{2}$ and $\:{pCO}_{2}$ denotes partial pressure. They are closely related and often scale together, but they are different physical quantities. $\:{fCO}_{2}$ is dimensionless in the equations and is converted to parts per million (ppm) by multiplying by 10^6^. We use $\:{pCO}_{2}$ when discussing the plotted atmospheric $\:{CO}_{2}$ values in ppm (Suppl. Figure 1,2); strictly, partial pressure and mixing ratio are proportional under fixed total atmospheric pressure, so the plotted $\:{pCO}_{2}$ values are the ppm representation of the model variable $\:{fCO}_{2}$. We use $\:{CO}_{2}$ more generally when referring to carbon dioxide as a chemical species, reservoir, or carbon-cycle process.

The choices *N* = 100 and T = 200,000 years were made for numerical sampling reasons. The Bayesian-mechanics readout estimates boundary-conditioned modes over a two-dimensional blanket-state grid and then fits a synchronisation map between internal and external conditional modes. Stable estimation therefore requires both many independent stochastic realisations and a long post-burn-in sample so that the relevant blanket-state bins are sufficiently populated. With dt = 0.1 years, these settings provide 2,000,000 time steps per realisation and 20,000,000 post-burn-in samples across the ensemble for conditional-mode estimation, while keeping the computation tractable.

**Table 1:**
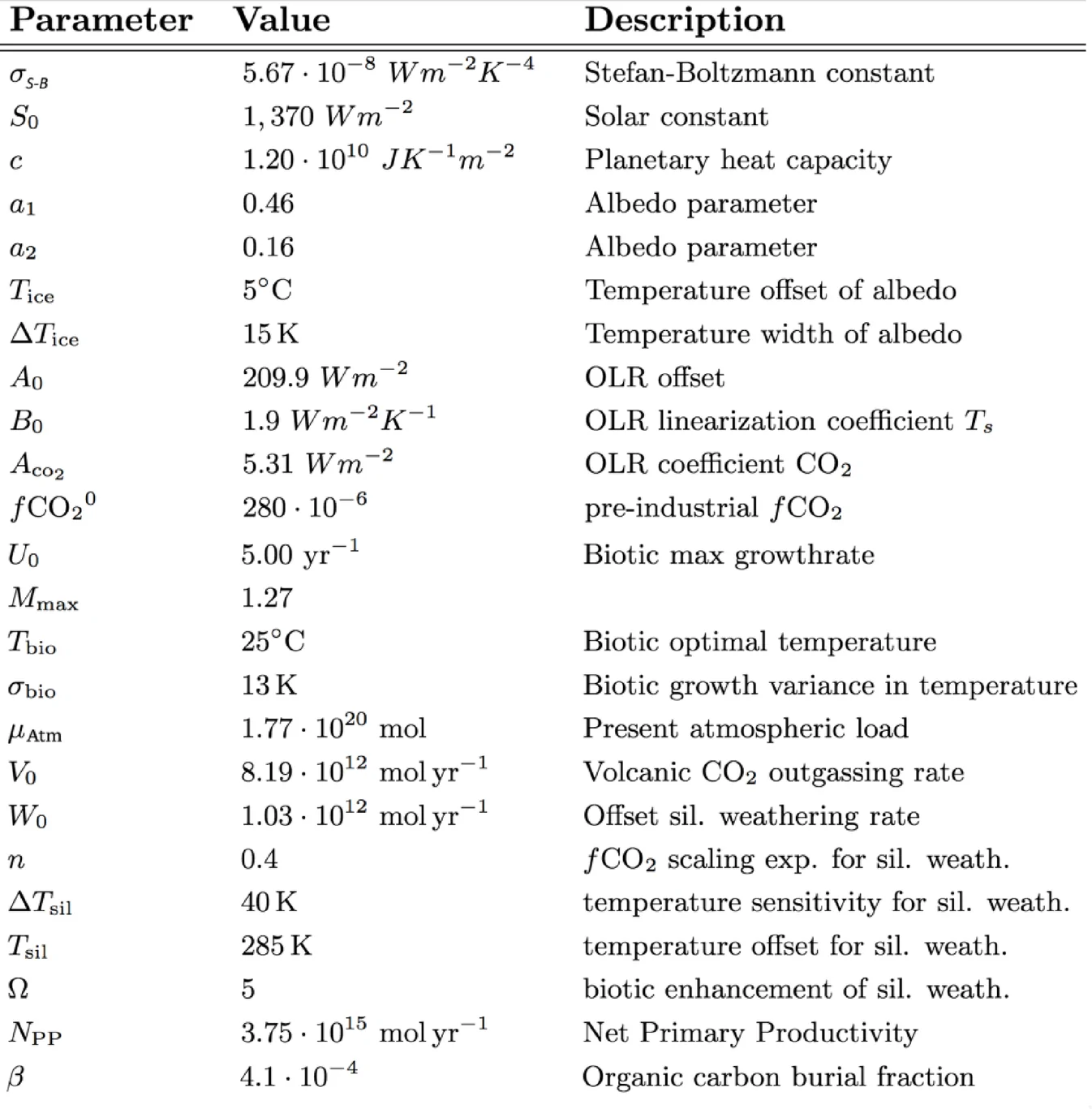
Model’s parameters and constants^37^.

### Pre-processing

#### Standardisation

In preparation for the Bayesian analysis, we normalised each variable ($\:{M}_{bio},\:fC{O}_{2\:},\:{T}_{s},\:{\:R}_{in}$) by centering and scaling each variable and by its respective time- and realisation-averaged mean and standard deviation. Note that this assumes the system is ergodic at the NESS (i.e., its time and ensemble statistics are approximately equivalent). This condition is satisfied because each variable is subject to random fluctuations.

### Estimating the synchronisation function

We empirically fit the synchronisation map to the conditional modes (the boundary-conditional most likely internal and external states) using linear regression^31^. In the absence of a closed-form expression for the system’s stationary probability distribution and thus expression for the conditionals, we need to estimate the modes of the conditional densities. Depending on the analysis, we did this one of two ways, which we outline below:

### Conditional mode estimation: Multi-dimensional histogram method

The first method for conditional mode estimation relies on generating discrete approximations to the conditional distributions over internal and external states, given blanket states, i.e., $\:P\left(\mu\:\right|b)$ and $\:P\left(\eta\:\right|b)$, respectively. We achieved this by first approximating the full joint distribution of the system at steady state with a multi-dimensional histogram, computed using samples from all independent stochastic realisations from the final $\:{10}^{5}$ timesteps of all stochastic realisations. Assuming ergodicity, the resulting time- and realisation-aggregated histogram can be treated as a discrete approximation to the joint probability of the system’s four variables at steady state. For histogram computation, we binned the data in each dimension with 150 non-overlapping, discrete and uniformly-sized bins; the particular choice of 150 for the number of bins was selected to balance the exponential scaling of N-dimensional histogram estimation with increasing bin number, with sufficiently detailed estimation of the synchronisation map. Having estimated the N-dimensional histogram approximation to the steady state, we can then estimate the conditional modes as follows: for all 2-dimensional bins of the blanket states (all possible $\:{150}^{2}$ combinations of bins of $\:{T}_{s}$ and $\:fC{O}_{2}$) we calculated the conditional modes $\:P\left(\eta\:\right|b)$, $\:P\left(\mu\:\right|b)$ as the bin of the internal (respectively, external) states that was assigned highest probability or count according to the approximated joint density. The result of this method is a pair of 2-D ‘images’ of discrete points (of resolution 150 × 150) that lie on the (approximated) conditional synchronisation manifold. The resulting pairs of images of conditional modes can then be used to fit the synchronisation function using regression, as described in detail below (see *Regression between the conditional modes*).

### Conditional mode estimation: Bin-conditional average

The second method for conditional mode estimation is based on an established approach^40^, where the steady-state joint distribution of only the blanket states is estimated using discrete histograms. In this case we once again used 150 bins, although in practice this can be increased much further, due to the computational advantage gained by binning only the marginal blanket distribution, as opposed to the whole system’s stationary density. Once this discrete joint histogram over blanket states is computed, then for each blanket bin (all possible $\:{150}^{2}$ combinations of bins of $\:{T}_{s}$ and $\:fC{O}_{2}$), we estimate the conditional mode of the internal (respectively external) states by computing the empirical mean of (internal or external) states that occurred concurrently to those blanket states that lie within a given bin. We used this second method to generate Fig. 2C, and in that case also computed the bin-conditional variance of all those internal states that occurred simultaneous to blanket states associated with a given bin.

### Regression between the conditional modes

Visual inspection of the joint space of the conditional modes, in terms of the boundary states, (which in this case is straightforward due to the low-dimensionality of the boundary state-space) suggests that the joint conditional modes lie on a plane; this inspired us to use ordinary least squares (OLS) regression to estimate a linear function that predicts the conditional external mode (b) from the conditional internal mode (b):

10 $${\begin{aligned}{\boldsymbol{\eta }}(b_{t} ) &= {\sigma} _{{est}} ({\boldsymbol{\mu }}(b_{t} )) \\ &= k_{1} \mu _{1} (b_{t} ) + k_{2} \mu _{2} (b_{t} ) + C_{1} \end{aligned}}$$

In other words, we calculated the coefficients $\:{k}_{1}$, $\:{k}_{2}$, $\:{C}_{1}$ that minimise the averaged squared difference between the ‘observed’ most-likely external state **η**($\:{b}_{t}$) and the ‘predicted‘ most likely external state $\:{\sigma\:}_{est}$(**µ**($\:{b}_{t}$)). The estimated synchronisation map $\:{\sigma\:}_{est}$ is entirely characterised by the linear model in Eq. (10) with model parameters $\:{k}_{1}$, $\:{k}_{2}$, $\:{C}_{1}$.

This approach yields an approximation of the synchronisation map by fitting a statistical model that predicts the (estimated) conditional modes of the external states from the (estimated) conditional modes of the internal states. Note that not only is the model itself an estimation and has its own bias and variance, but the conditional modes themselves must also be estimated (e.g., according to the methods described in the previous section).

For estimating the conditional variances along with the conditional modes (as shown in Fig. 2C, for example), we fit an additional linear model to predict the *variance* of the boundary-conditional external state distribution, using the boundary-conditional internal mode, denoted $\:\varsigma\:\left({\mu\:}_{t}\right)$. This second synchronisation function $\:{{\varsigma\:}}_{est}$ maps from the most likely internal state **µ**(b) to the precision of the posterior belief p$\:\left(\eta\:\right|b)$ around the conditional mode **η**(b), i.e., the inverse of $\: - \nabla \:_{{\eta \:}}^{2} logp\left( {\eta \:|b} \right)_{{\eta \: = \boldsymbol{\eta} \:\left( b \right)}}$. This model was fit entirely independently of the first model, but still considered a function of the boundary-conditional internal mode. This follows as, when characterising Gaussian (i.e., Laplace) approximations to posterior beliefs, the conditional variance is a function of the boundary states and boundary-conditional internal mode^73^, however since the map * b* $\:\to\:$**µ**(* b*) in our model is injective, the conditional variance can be recovered solely using the boundary-conditional internal mode. As with the synchronisation function applied to the internal modes, this synchronisation map for the variances is characterised by a set of linear coefficients $\:{k}_{3}$, $\:{k}_{4}$,$\:\:{C}_{2}$ that predict a conditional external variance as a function of the internal states.

Estimating both synchronisation maps $\:\left({\sigma\:}_{est}\right)\:$allows us to reformulate the dynamics of the biosphere-climate system in terms of active inference. Specifically, this results from applying the synchronisation function $\:{\sigma\:}_{est}$ to an actual trajectory of internal states ($\:{\mu\:,\:M}_{bio}$), to furnish a continuous and accurate prediction of external states ($\:\eta\:$, $\:{R}_{in}$). Formally, this inference is upon an evolving Bayesian posterior belief about the current value of external states given the current value of boundary states:

11 $$q_{\mu } (\eta ) = N\left( {\eta ;\sigma \left( \mu \right),\varsigma \left( \mu \right)} \right)$$

Where $\:{q}_{\mu\:}$($\:\eta\:$) is the actual belief, $\:\sigma\:\left(\mu\:\right)\:$is a conditional mean and $\:\varsigma\:\left(\mu\:\right)\:$a conditional covariance (inverse precision) that predicts external states as a continuously-evolving Gaussian with mode $\:\sigma\:\left(\mu\:\right)$ and variance $\:\varsigma\:\left(\mu\:\right).$.

Because the synchronisation map is a function that maps the most likely value of the internal conditional density p(µ|b) to the most likely value of the external posterior belief (conditional density p(η | b)), the inference will only be exact at the time points where both the internal and external states assume their most likely values, given the current boundary state $\:{b}_{t}$. That is when $\:{\mu\:}_{t}=$ **µ**($\:{b}_{t}$) and $\:{\eta\:}_{t}=$ **η**($\:{b}_{t}$). Another reading of this approximation is that the NESS density is associated with a random attractor that is locally Gaussian (i.e., the Laplace approximation holds)^74^^75^,.

### Falsification control experiments

We have implemented a falsification control analysis in which the biosphere ($\:{M}_{bio}$) is either frozen (depleted/extinct) or decoupled from the climate-carbon variables. For this the internal state ($\:{M}_{bio}$) was retained as a stochastic state but made dynamically independent of the climate-carbon variables. Specifically, $\:{M}_{bio}$ evolves as an autonomous stochastic process, while biomass dependence is removed from the atmospheric $\:{CO}_{2}$ Eq. 7 by eliminating the biomass-enhanced weathering (Ω = 0) and biomass-dependent burial $\:{(\beta\:}_{NNP}=0)\:$terms. The four-variable state space and the same Markov-blanket partition are retained, so the comparison tests the role of biological coupling for the inference of the system.

### Sensitivity and robustness experiments with a simplified and augmented model

After the falsification control experiments, additional simplification of the $\:{CO}_{2}$ Eq. 7 was performed because this is where most of the biological coupling enters the baseline biosphere-climate system model. Starting from the baseline model, we examined: removal of the biomass enhancement of silicate weathering (Ω = 0), removal of the biomass-dependent organic burial sink $\:{(\beta\:}_{NNP}=0),\:$removal of both biomass-dependent $\:{CO}_{2}$ sink (Ω = 0, $\:{\beta\:}_{NNP}=0)$ terms and changes to the silicate-weathering $\:{CO}_{2}$ exponent from baseline model value *n* = 0.4 toward a linear dependence (*n* = 0.7 and *n* = 1.0), and the independent-biomass / decoupled-carbon-cycle control described above. All cases were analysed with the same Bayesian mechanics analysis explained above, studying the map between the blanket-conditional modes of the internal and external states.

### Perturbation experiments

Each perturbed trajectory was independently generated from a historical simulation, rather than starting all “perturbed trajectories” (starting at time point 0) from the same starting state. In other words, the only thing that relates the perturbed trajectories to each other is their starting value of $\:{T}_{s}$ – their other starting values (of $\:{R}_{in}$, $\:{fCO}_{2}$, $\:{M}_{bio}$) are sampled from the stationary distribution.

We performed the analysis assuming $\:{R}_{in}$ as a dynamical relaxing variable. In the perturbation set of analyses we showcase predictive processing for the stochastic biosphere-climate dynamical system by subjecting the system to fixed sensory perturbations.

### Robustness analysis

We calculated the synchronisation map correlations by sweeping up to $\:{S}_{0}$ = 1400–1650 $\:W{m}^{-2}$. We did a sweep around the default value from $\:{S}_{0}$ = 1360–1380 $\:W{m}^{-2}$ in steps of 1.0 $\:W{m}^{-2}$.

### Simulation software

The Python code for running the simulations and the Bayesian mechanics analysis can be accessed at the following GitHub repository: https://github.com/conorheins/climate_fep.

## Supplementary Information

Below is the link to the electronic supplementary material.

Supplementary Material 1

## Data Availability

The datasets generated and analyzed during this study, necessary to evaluate the article’s conclusions and replicate the experiments, can be reproduced using the `climate_fep` repository on GitHub, available at the following link: [https://github.com/conorheins/climate_fep](https:/github.com/conorheins/climate_fep) by requesting it from the corresponding author.
