# Supplementary material for: An elementary form of agency at planetary-scale
Source: Sci Rep. 2026 Aug 3;16:35838. doi: 10.1038/s41598-026-61878-9 (PMC13719523; doi:10.1038/s41598-026-61878-9)
Supplement: Supplementary file 1 [file 41598_2026_61878_MOESM1_ESM.docx]

**Supplementary Information**

We investigated the relatively large fluctuations in $\boldsymbol{p}CO_{2}$ in Figure 2A (main manuscript) by running a six-case mechanism analysis on the stochastic biosphere-climate model under the same baseline settings as the Figure 2C-style analysis, comparing:

i) the baseline stochastic system,

ii) a fully deterministic system,

iii) a system with biomass noise removed,

iv) a system with the biomass-dependent weathering term removed (Ω = 0$)$

v) a system with the biomass-dependent burial term removed $(\beta_{NNP}=0)$, and

vi) a system with both (Ω = 0, $\beta_{NNP}=0)$biomass-dependent $CO_{2}$ sink terms removed.

The main result is that the large $\boldsymbol{p}CO_{2}$ fluctuations are driven primarily by stochastic forcing in the original SDE formulation. In the baseline stochastic system, $\boldsymbol{p}CO_{2}$ fluctuates around a bounded post-burn-in regime (Supplementary Fig. 1A), and the post-burn-in $\boldsymbol{p}CO_{2}$ standard deviation is 6.08 ppm. In the deterministic version, the corresponding standard deviation collapses to 0.00 ppm (Supplementary Fig. 1A,C). By contrast, removing biomass noise alone has almost no effect on the fluctuations (6.06 ppm; Supplementary Fig. 1C), which indicates that the large $\boldsymbol{p}CO_{2}$ variability is not specifically caused by stochasticity in the biomass equation itself. The biomass-dependent carbon-cycle couplings instead act mainly as stabilizing feedbacks on the mean carbon state. This is visible in the near-cancellation of the source, weathering, and burial terms in the baseline $\boldsymbol{f}CO_{2}$ equation (Supplementary Fig. 1B). Removing the biotic weathering term raises the mean $\boldsymbol{p}CO_{2}$ from 179 ppm to 3691 ppm and increases its standard deviation to 76.79 ppm, while removing both biomass-dependent sink terms raises the mean to 4976 ppm and the standard deviation to 107.44 ppm (Supplementary Fig. 1C).

**Supplementary Figure 1**. **Mechanism analysis of the relatively large** $\boldsymbol{p}CO_{2}$ **fluctuations in the stochastic biosphere-climate model**. **A**) Representative post-burn-in $\boldsymbol{p}CO_{2}$ traces for the baseline stochastic model and the deterministic version. The stochastic run fluctuates around a bounded regime, whereas the deterministic run does not show comparable variability. **B**) Signed contributing terms in the baseline $\boldsymbol{f}CO_{2}$ equation. The source term, silicate-weathering sink, and biomass-dependent burial sink nearly balance, leaving a small net drift; this shows that the deterministic $CO_{2}$ terms primarily stabilise the mean carbon state. **C**) Post-burn-in $\boldsymbol{p}CO_{2}$ standard deviation across the six mechanism cases. The deterministic case collapses to zero variability, biomass-noise removal has little effect, and removal of biomass-dependent $CO_{2}$ sink terms greatly increases variability. **D**) Lagged mean-squared displacement of $\boldsymbol{p}CO_{2}$. Mean squared displacement (MSD) grows with lag because finite-lag stochastic fluctuations accumulate in a weakly mean-reverting system. The much larger MSD in the Ω = 0 and $\beta_{NNP}=0$conditions reflects their shifted high-$CO_{2}$ regimes and longer correlation times, explaining their larger $\boldsymbol{p}CO_{2}$ variability without implying that the baseline $\boldsymbol{p}CO_{2}$ dynamics are an unbounded random walk.

**Supplementary Figure 2.** $\boldsymbol{pCO}_{\boldsymbol{2}}$**relaxation detail.** Long-horizon ${pCO}_{2}$recovery after perturbation (see Figure 3A top left panel). The black curve shows ensemble-mean ${pCO}_{2}$, grey shading shows (± 1) standard deviation, and the green line/band show the pre-perturbation mean and (± 1) pre-perturbation standard deviation. Over 5000 years, ${pCO}_{2}$remains within the baseline stochastic band and returns close to its pre-perturbation mean; the final 500-year mean differs from baseline by only -0.27 ppm. A stricter ± 1 ppm sustained-return criterion is reached after approximately 2615 years.

In the present work, we employed the solar constant value of S_0_ = 1370 $Wm^{-2}$of the deterministic biosphere-climate model, which, indeed, is likely based on the old observations in 1980-1990. The currently-accepted value of the solar constant based on recent observations is close to 1361 $Wm^{-2}$(Kopp, 2025).

We therefore performed an additional sensitivity analysis comparing the value S_0_ = 1370 $Wm^{-2}$ with S_0_ = 1360 $Wm^{-2}$, while keeping the rest of the model and analysis pipeline unchanged. The inference-like synchronisation result is not weakened by using the modern solar constant value. With S_0_ = 1370 $Wm^{-2}$ , the mean inferred-vs-actual $R_{in}$ correlation across trajectories was 0.876 and the fitted sync-map slope was 4.09. With S_0_ = 1360 $Wm^{-2}$, the mean inferred-vs-actual Rin correlation was 0.895 and the fitted sync-map slope was 3.62. We therefore retain S_0_ = 1370 $Wm^{-2}$in order to remain faithful to the deterministic biosphere-climate model parameterization, but note that the main result is robust to replacing it with the current observed value.

**Supplementary Figure 3. Sensitivity analysis to the realistic changes in solar constant value. A**) Mean inferred-vs-actual $R_{in}$ correlation across trajectories for the baseline biosphere-climate system model S_0_ = 1370 $Wm^{-2}$value and the current S_0_ = 1360 $Wm^{-2}$value. **B**) Fitted conditional-mode sync-map slopes for the same two cases. **C-D)** Representative inferred and actual Rin traces. The current solar constant value S_0_ = 1360 $Wm^{-2}$does not reduce the active inference and synchronisation signature, supporting the use of the original value for consistency with the source model.

**Supplementary Figure 4**. **Sensitivity of the synchronisation and active inference result to simplifications of the atmospheric** ${CO}_{2}$ **equation and to the independent-biomass decoupling control in the baseline biosphere-climate system model.** **A)** Mean inferred-vs-actual $R_{in}$ correlation across trajectories. **B)** Fitted conditional-mode sync-map slope. **C-G)** Representative inferred and actual $R_{in}$ trajectories for the baseline model, removal of the biomass-dependent burial sink ($\beta_{NNP}= 0$), removal of biomass-enhanced silicate weathering $\Omega$= 0, linear silicate-weathering ${CO}_{2}$ dependence (n = 1.0), and independently evolving but dynamically decoupled biomass. The baseline manuscript equation gives the strongest overall inferred-vs-actual $R_{in}$ prediction performance. The $\Omega= 0$, $\beta_{NNP}= 0$ case retains a fitted synchronisation map in conditional-mode space, but its prediction performance is degraded and the fitted slope reverses sign, so it is not interpreted as preserving the same active-inference-like relation. The independent-biomass decoupling control provides the clearest falsification: biomass remains stochastic, but because it is no longer coupled through the carbon-cycle feedbacks, the inferred $R_{in}$ trace becomes essentially flat and the inferred-vs-actual correlation collapses.

**Supplementary Figure 5**: **Two-biomass augmented model.** **A)** Representative trajectory of the augmented five-dimensional system in which the original single biomass state is replaced by two biomass variables with different optimal temperatures, while surface temperature, atmospheric $CO_{2}$, and incoming radiation remain shared. The plotted biomass traces show that the two biological components occupy distinct dynamical ranges rather than simply duplicating the original biomass state. **B)** Inferred and actual $R_{in}$ trace obtained from the conditional-mode synchronisation map. The strong inferred-vs-actual correlation (0.951) and sync-map R2 (0.819) show that the inference-like internal-external relation is preserved when the biological sector is expanded from one biomass variable to two biomass variables.

We examined a latitudinally resolved 7-dimensional stochastic biosphere-climate model with separate high- and low-latitude temperature, biomass, and incoming-radiation variables, coupled through a shared atmospheric $CO_{2}$ state. The top panels of the figure show a representative trajectory of this augmented system (Supplementary Fig. 6).

In this 7-dimensional version, the inference synchronisation result is still present, but it is weaker than in the 4 and 5-variable model. In the analysis shown here, where the conditional mapping is estimated by conditioning on the sensory states and using a median-based estimator, the representative trajectory gives an inferred-vs-actual correlation of 0.450 for the high-latitude $R_{in}$ variable and 0.042 for the low-latitude $R_{in}$ variable, with an overall multivariate sync-map R2 of 0.528 (Supplementary Fig. 6).

We therefore interpret the 7D model as a robustness test that gives preservation of the original result in a more structured latitudinal model, rather than a full reproduction of the strong Figure 2C-style tracking seen in the original four-variable system. This analysis serves as evidence that increasing dimensionality doesn't break the Bayesian-mechanical behaviour of the resulting system (Supplementary Fig. 6).

**Supplementary Figure 6:** **Latitudinally resolved seven-dimensional stochastic biosphere-climate model. A**) Representative trajectory from the augmented model with high- and low-latitude temperature, biomass, and incoming-radiation variables coupled through a shared atmospheric $CO_{2}$ state. The trajectory shows the intended two-box structure: warmer low-latitude temperature, cooler high-latitude temperature, separate biomass components, and bounded shared $CO_{2}$. **B**) Figure 2C-style inferred and actual incoming-radiation traces for the high- and low-latitude external variables. The high-latitude $R_{in}$ variable retains a moderate inference-like synchronisation relation (correlation 0.450), while the low-latitude $R_{in}$ variable is much weaker (correlation 0.042), with multivariate sync-map R2 = 0.528. This indicates partial preservation, but not full reproduction, of the original four-variable result under a more structured augmented model.

**Supplementary Figure 7.** **Sensitivity analysis of the seven-dimensional latitudinal model after changing the optimal biomass temperatures to** $\boldsymbol{T}_{\boldsymbol{bio\_high}}$ **= 16°C and** $\boldsymbol{T}_{\boldsymbol{bio\_low}}$ **= 22°C.** **A**) Representative trajectory of the augmented system under this parameterization. **B**) Inferred and actual high- and low-latitude $R_{in}$ traces (top and bottom panels respectively) from the sensory-conditioned median conditional-mode analysis. Lowering the low-latitude biomass optimum improves the low-latitude inferred-vs-actual correlation relative to the default 7D parameterization, while preserving a moderate high-latitude relation and increasing the multivariate sync-map R2.
